# Nurses’ Perceptions of Clinical Education for Nursing Students in Japan During the COVID-19 Pandemic: A Cross-Sectional Study

**DOI:** 10.7759/cureus.78658

**Published:** 2025-02-06

**Authors:** Rika Soeda, Hiroko Kumai, Ayako Suzuka, Megumi Kamogawa, Sumiyo Ota, Shun Yoshihara, Hideaki Sakuramoto, Kohei Kajiwara, Junko Shiraki, Yuka Yamamoto, Kimie Harada

**Affiliations:** 1 Faculty of Nursing, Japanese Red Cross Kyushu International College of Nursing, Munakata, JPN; 2 Department of Nursing, Imazu Red Cross Hospital, Fukuoka, JPN; 3 Department of Nursing, Kama Red Cross Hospital, Kama, JPN

**Keywords:** clinical practice, covid-19, nursing education, survey, web questionnaire

## Abstract

The COVID-19 pandemic has affected healthcare workers and medical students including the restriction of nursing students from clinical practice, which hinders the learning process. This study examined changes in nurses’ perceptions concerning nursing students’ clinical practice of education during the COVID-19 pandemic.A web-based questionnaire was administered to nurses from two facilities, which comprised pre- and post-pandemic teaching experiences. IBM SPSS Statistics for Windows, Version 26.0 (Released 2019; IBM Corp., Armonk, NY, USA) and KH Coder (KH Coder 3.0, Koichi Higuchi, Tokyo, Japan) were utilized for the data analysis in this study. Among the 119 questionnaires distributed, 53 were analyzed (77.9% valid response rate). Overall, 43 (92.5%), 38 (71.7%), and 44 (83.0%) respondents perceived changes in nursing students’ clinical practice, nursing students, and their own teaching practices during the COVID-19 pandemic, respectively.Many nurses perceived reduced patient interaction and shifts in student characteristics during clinical practice. Nurses reacted to these changes by confirming students’ understanding and asking more questions. The limited sample size and cross-sectional design suggest the need for further exploration of the construct’s concept and model. The findings highlight the importance of supporting nursing educators in adapting their teaching methods to address COVID-19 pandemic challenges.

## Introduction

Clinical practice has always been necessary for nurse training. Many studies have been conducted on the clinical practice of nursing students [1,2]. However, the COVID-19 pandemic has substantially affected healthcare workers and medical students. At the peak of the pandemic, nursing students were restricted from clinical practice, which is an important learning process.

Studies have reported the effects of the COVID-19 pandemic on the mental health and life satisfaction of nursing students [3,4]. Several reviews have systematically organized research on clinical practice, including clinical judgment and patient relationships [5,6]. A review of nursing students amidst the COVID-19 crisis highlighted the utilization of simulation as an alternative to clinical practice, alongside the continuation of clinical training and its associated challenges [7]. The transformation of nursing education during other coronavirus pandemics has been documented, emphasizing the efficacy of the COMFORT COVID-19 communication modules and the potential of competency-based online education [8]. Among them, reports on the impact of COVID-19 and the effectiveness of simulation education have emerged. The reduced opportunities for nursing students to practice directly with patients and learn teamwork should be considered regarding their impact on the education of newcomers in post-graduate clinical practice, which has been restricted by COVID-19.

Limitations attributed to the COVID-19 impact have also been highlighted [7]. However, only a few studies have presented methods to provide better clinical practice opportunities for students affected by the pandemic within clinical settings. Additionally, the effect of the pandemic on clinical practice based on the perception of faculty members was reported [9]; the perceptions of nurses at hospitals who supervise clinical education have also been described.

Simulation education and other activities have made great progress under the COVID-19 pandemic [10]. Even in the absence of clinical practice, the environment in which nursing could be learned has been greatly advanced. However, interpersonal restrictions have reduced the communication skills of many people, including younger people [8]. Therefore, after experiencing a global pandemic of emerging infectious diseases, the realistic learning experience of clinical practice needs to be reexamined. In clinical practice settings, nurses play a pivotal role in education, making it imperative to focus on their perceptions.

An understanding of changes in practical training instructions provided to nursing students based on the perceptions of nurses who provide this education is necessary to determine the support required to improve clinical practice in an era of emerging infectious diseases. These previous findings indicate the need to obtain suggestions regarding the nurses’ education, who play an important role in practice after a global pandemic of an infectious disease. This study aimed to explore the changes in nurses’ perceptions of clinical practice education for nursing students during the COVID-19 pandemic.

## Materials and methods

Participants

Registered nurses at two facilities who provided clinical education to nursing students before and after the COVID-19 pandemic in Japan participated. The facilities were selected through snowball sampling. Given the study’s preliminary nature, this sampling method was deemed appropriate. The nursing directors of two facilities that had accepted clinical education to nursing students before and after the COVID-19 pandemic were asked to participate in the survey. A survey questionnaire was randomized from the nursing directors of the facilities to registered nurses who provided clinical education to nursing students before (prior to March 2020) and after the COVID-19 pandemic.

Data collection

The participants answered a web-based questionnaire from February to March 2023. This study used a cross-sectional study.

Measurements

The questionnaire was formulated following a comprehensive review by the research team. As a preliminary survey, it was designed with a succinct item structure to facilitate the exploration of relevant factors. The questionnaire included items regarding how long the nurses had been practicing, their experience as clinical practice supervisors, and their experience in clinical practice supervision. Questionnaire items were developed by the researcher. The questionnaire included items regarding how long the nurses had been practicing, their experience as clinical practice supervisors, and their experience in clinical practice supervision. The following questions were asked: “Have you perceived any changes in clinical practice during the COVID-19 pandemic compared to before the COVID-19 pandemic?,” “Do you perceive that nursing students during the COVID-19 pandemic have changed compared to nursing students before the COVID-19 pandemic?,” and “Do you recognize that your own teaching practice instructions during the COVID-19 pandemic have changed compared to before the COVID-19 pandemic?”

Data analyses

Data on nurses’ attributes and perceptions of changes in clinical practice were analyzed using descriptive statistics, with quantitative data processed via IBM SPSS Statistics for Windows, Version 26.0 (Released 2019; IBM Corp., Armonk, NY, USA). We conducted a comprehensive analysis of the qualitative data using a powerful and widely recognized text-mining method called KH Coder (KH Coder 3.0, Koichi Higuchi, Tokyo, Japan; http://khcoder.net/en) [11]. Frequently occurring words were extracted from the free descriptions, and their co-occurrence networks were analyzed. The minimum number of occurrences after the extraction was set at three.

Ethics approval statement

The study was approved by the Ethics Committee of the Japanese Red Cross Kyushu International College of Nursing (approval number 22-021). All participants consented to their participation in the study. This study was conducted in accordance with the tenets of the Declaration of Helsinki.

## Results

The questionnaire was distributed to 119 nurses at two participating institutions, and responses were obtained from 68 participants. We analyzed the responses of 53 nurses who had experience in training both before and after the COVID-19 pandemic (valid response rate: 77.9%). The participants’ demographic characteristics are presented in Table 1. The median for years of experience as a nurse was 22.0 (IQR: 13.5-28.0) years, and the median for years of experience as a nurse at the current hospital was 12.0 years (13.5-17.5). Forty-three participants (81.1%) had clinical training instructor experience.

**Table 1:** Demographic characteristics of participants

	N = 53
Years of experience as a nurse	22.0 (13.5-28.0)
Years of experience at the current hospital	12.0 (9.0-17.5)
Experience as an on-site training instructor	43 (81.1)

A total of 906 words were extracted using the KH Coder from the free-text responses regarding the changes in teaching clinical practices (Table 2). Of the 147 words analyzed, the types of words extracted were 219. A total of 836 words were extracted regarding the changes in nursing students. Of the 163 words analyzed, the types of words extracted were 232. A total of 1,082 words were extracted regarding personal changes in teaching practice instructions. Of the 201 words analyzed, the types of words extracted were 285.

**Table 2:** Frequent words in free-text survey responses

Changes regarding on-site training		Changes regarding nursing students		Changes regarding training instruction methods	
Extracted word	Number of appearances	Extracted word	Number of appearances	Extracted word	Number of appearances
Patient	31	Patient	19	Practical training	15
Practical training	22	Student	14	Student	14
Time	16	Practical training	13	Infection	12
Field trip	12	Communication	11	Instruction	12
Student	11	Feel	11	Patient	9
Restriction	11	Few	10	Explanation	9
Infection	9	Proactive	8	Countermeasure	8
Be involved	8	Many	8	Field trip	7
Decrease	7	Involvement	6	Learn	6
Countermeasure	7	Increase	6	Experience	6
Care	6	Be involved	5	Consider	6
Involvement	6	Field trip	5	Time	6
Touch	6	Time	5	Clinical	6
Opportunity	5	Before	4	Practice	5
Direct	5	Study	4	Many	5
Difficult	5	Posture	4	Care	4
Nursing	4	Actual	4	Consciousness	4
Few	4	Decline	4	Involvement	4
Contact	4	Clinical	4	Think	4
Short	4	Side	3	Perform	4
Shortening	4	Nursing	3	Few	4
Communication	3	Consider	3	Note	4
Shield	3	Take	3	Inquire	4
Face	3	Touch	3	Understanding	4
Family	3	Development	3	Communication	3
Conversation	3	Compare	3	Before	3
Feel	3	Change	3	Confirmation	3
Decrease	3			Be involved	3
Confusion	3			Opportunity	3
Can perform	3			Question	3
Think	3			Actual	3
Instruction	3			Touch	3
Can do	3				

**Figure 1:**
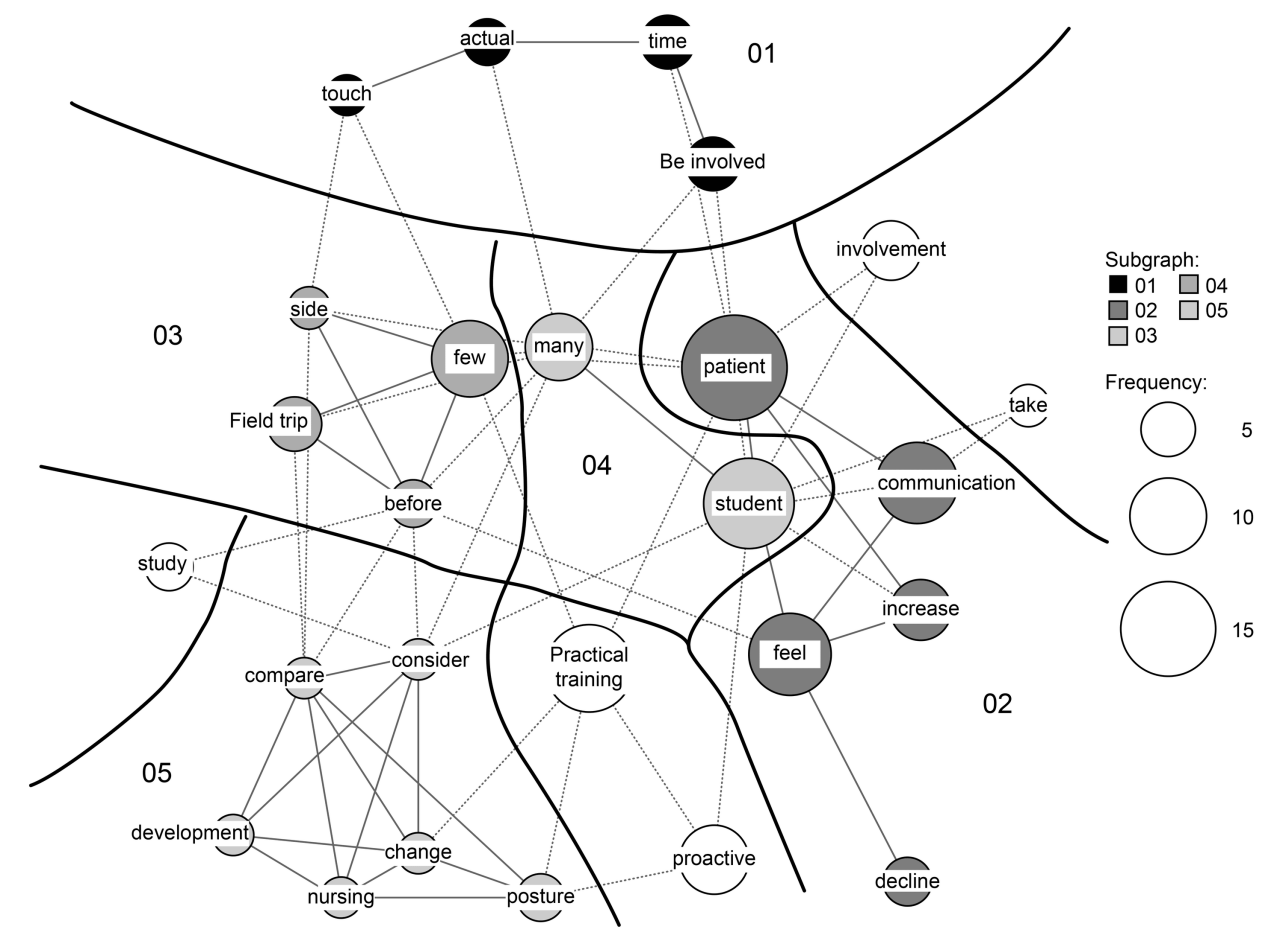
Co-occurrence network regarding changes in nursing students The size of the circle represents the number of occurrences of a word and the subgraphs (the word within nurses’ perceptions) are divided into strongly connected groups. The size of the circle represents the number of occurrences of a word and the subgraphs are divided into strongly connected groups. The lines connecting the circles represent co-occurrence relationships, with the same group of subgraphs connected by solid lines and different groups connected by dotted lines.

Five subgraphs were represented in the co-occurrence network for changes in the nursing students (Figure 1). In subgraph 3, the words “few,” “field trip,” “before,” and “side” were linked, and “actual,” “touch,” “be involved,” and “time” were linked in subgraph 1. “Nursing,” “development,” “compare,” “change,” “posture,” and “consider” were linked in subgraph 5. The words “students,” “many,” “patients,” “communication,” “increase,” and “feel” were linked in subgraph 4, and “feel” and “decline" were linked in subgraph 2.

**Figure 2:**
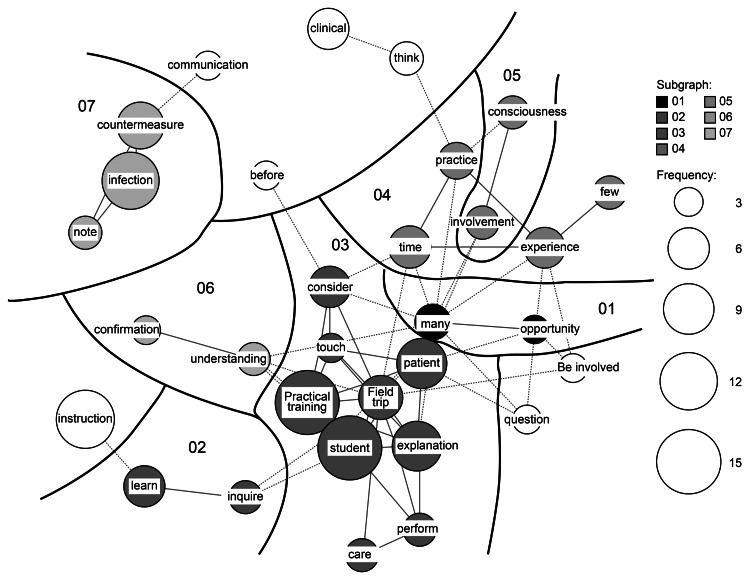
Co-occurrence network regarding changes in training instruction methods The size of the circle represents the number of occurrences of a word and the subgraphs (the word within nurses’ perceptions) are divided into strongly connected groups. The lines connecting the circles represent co-occurrence relationships, with the same group of subgraphs connected by solid lines and different groups connected by dotted lines.

Seven subgraphs were represented in the co-occurrence network regarding the changes in teaching practice instructions (Figure 2). In subgraph 7, “note” and “countermeasure” were independently categorized around “infection.” “Student” and “practical training” were linked to the words “patient,” “explanation,” and “field trip” in subgraph 3. In subgraph 4, “practice,” “experience,” “time,” and “few” were linked. In subgraph 2, “inquire” and “learn” were linked. In subgraph 6, “understanding” and “confirmation” were linked.

## Discussion

Based on the survey results, nurses recognized changes in clinical practice opportunities for nursing students, their teaching methods, and student characteristics since the COVID-19 pandemic. They perceived the nursing students had a decrease in opportunities to interact with patients due to limited training time and increased field trips, and they were unsure how to communicate and interact with patients.

The participants perceived a decline in the nursing students’ communication skills and positive attitudes. The pandemic restricted face-to-face learning opportunities, and students experienced several learning difficulties, including decreased motivation and concentration [12]. The students lacked confidence in their communication skills and could not actively engage with patients and other nurses owing to reduced interaction opportunities during the pandemic. Similar trends have been observed concerning clinical practice [7].

Limitations in simulation education were also identified, emphasizing the need for support in this area, given the potential for future epidemics of emerging infectious diseases. In addition, the training of new nurses and nursing students has changed from group to decentralized after the COVID-19 pandemic [13]. The educational format has also shifted to a hybrid format, such as simulation education, which has shown positive results [14]. Issues with communication skills can only be experienced in realistic communication situations, and the necessity of opportunities for hands-on training in a face-to-face setting should be revisited.

The participants also reported changes in their clinical teaching instructions as a response to changes in the clinical practice opportunities for the students. Those with practical training experience reported providing opportunities for observation, asking as many questions as possible, and checking for understanding to ensure that the students were learning from clinical situations, especially as practice time was limited and patient interactions were restricted during the pandemic. The participants aimed to reduce the gap between what students imagined and the clinical situation. The results also suggested that the participants were flexible in changing their teaching methods to meet the challenges of students' communication skills. A previous study reported the challenges of transitioning from theoretical knowledge to practice [15]. Moreover, the cultural context also constitutes a significant factor influencing nursing practice. The cultural context serves as a pivotal determinant in shaping nursing practice. Research comparing mentoring practices in Japan with those in other nations has highlighted notable disparities in mentoring competencies and educational backgrounds [16]. Acknowledging both the cultural context and the impact of the COVID-19 pandemic is crucial in providing effective support.

In this study, practice supervisors were involved in addressing such challenges during the COVID-19 pandemic, aiming to integrate practical experiences with theoretical knowledge gained from lectures and pre-practice learning situations. Given these factors, we believe that the importance of flexibility in a student’s situation is suggested by the fact that, even among educational institutions other than the on-site. We believe it is necessary to collaborate between basic education institutions and clinical settings such as hospitals and share information on educational methods that flexibly respond to students' learning environments. Therefore, future attempts to support clinical nurses to be more flexible in handling diverse nursing students will be necessary.

Limitations

This study is not without its limitations. First, the sample size was limited due to the preliminary study design, and based on the results, it is necessary to examine the concept and model of the construct and the utilization of a cross-sectional study design. Second, since this study was a self-administered survey, there could be self-reporting bias and recall bias. Third, this study pertained to a specific country context, whereas cultural trends can exert a significant influence on clinical practice. However, this study indicated nurses' perceptions of clinical practice after the COVID-19 pandemic, which will have implications for the education of future nursing students and for the development of how the nursing profession responds in emergency situations. We contend that augmenting the sample size and selecting items informed by the findings of this survey will contribute significantly to future research endeavors. In addition, we believe that it may connect to clinical practical education for post-graduate nurses.

## Conclusions

Nursing instructors reported changes in clinical practice opportunities for nursing students owing to the COVID-19 pandemic. Nursing students experienced a reduction in opportunities to engage directly with patients, attributed to shortened training periods and an increase in field trips, leaving them uncertain about effective methods of communication and interaction with patients. Moreover, the participants observed a deterioration in the nursing students' communication abilities and their display of a positive attitude. Nursing instructors reported the nursing students had decreased engagement with patients and changes in the students’ characteristics. Consequently, the instructors responded to such changes by examining students’ understanding and asking more questions.
